# Is Percutaneous Kyphoplasty the Better Choice for Treatment of Stage III Kümmell's Disease Without Neurological Symptoms? A Retrospective Study of Two Invasive Procedures

**DOI:** 10.1111/os.14313

**Published:** 2024-12-16

**Authors:** Yijie Liu, Tangyiheng Chen, Haoyun Yu, Xiaohui Zhou, Runjia Hua, Yudong Wang, Qiang Wei, Yong Gu, Genglei Chu

**Affiliations:** ^1^ Department of Orthopaedic Surgery The First Affiliated Hospital of Soochow University Suzhou Jiangsu China; ^2^ Department of Orthopaedic Surgery The Fourth Affiliated Hospital of Soochow University Suzhou Jiangsu China; ^3^ Medical College Soochow University Suzhou Jiangsu China; ^4^ Department of Orthopedics The First Affiliated Hospital of Anhui Medical University Hefei Anhui China

**Keywords:** body position resetting, Kümmell's disease, long‐segment fixation, pedicle subtraction osteotomy, vertebroplasty

## Abstract

**Study Design:**

Retrospective analysis.

**Objective:**

Kümmell's disease is an uncommon and complicated spinal condition first described in 1891. Treatment of this disease must be individualized according to the stage of disease and the experience and preference of the surgeon. Nevertheless, the surgical option in Stage III Kümmell's disease without neurological deficits remains controversial. The purpose of this study is to determine whether PKP or pedicle subtraction osteotomy (PSO) combined with long‐segment fixation (LSF) is more effective in treating Kümmell's disease at Stage III without neurological impairments.

**Methods:**

Between January 2017 and June 2020, 89 patients were treated with PKP or PSO + LSF. The outcomes, including operative time, blood loss, Oswestry Disability Indexes (ODIs), heights of fractured vertebrae, visual analog scale (VAS) scores, and kyphosis Cobb angles, were measured at the follow‐up time for the PKP group and PSO + LSF group. Fisher's exact test or chi‐square test for number and percentage data was employed to compare statistical analyses between two groups.

**Results:**

Forty‐six patients underwent PKP and 43 patients who treated by PSO + LSF. Postoperative measurements showed substantial improvements in kyphosis Cobb angle and vertebral height in the PKP group compared to preoperative measurements. Operating time, estimated blood loss, and duration of stay were all reduced in the PKP group compared to the PSO + LSF group. The PSO + LSF group had better correction of a kyphotic Cobb angle than the PKP group. Short‐term monitoring showed that the PKP group had fewer ODI and VAS scores than the PSO + LSF group. In addition, no significant neurological symptoms were found after operation in both groups. The complication rates of PKP and PSO + LSF groups were 10.87% and 9.30%, respectively.

**Conclusions:**

Kümmell's disease in Stage III without neurological symptoms responded to both PKP and PSO + LSF as safe and efficient treatments. Despite limited correction of kyphotic Cobb angle, PKP patients had better early clinical outcomes, increased fractured vertebral height, decreased blood loss, and less surgical trauma compared with the PSO + LSF group.

## Graphical abstract

Kümmell's disease occurs more frequently after OVCF with vascular osteonecrosis of vertebral body. Based on the amount of clinical practice, we considered PKP as a feasible treatment for Stage III Kümmell's disease without neurological symptoms.

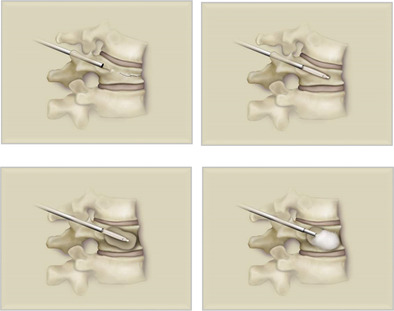

## 1 Introduction

Kümmell's disease is a subtype of osteoporotic vertebral compression fracture (OVCF), and it is becoming increasingly common, particularly given the rising number of OVCF cases caused by osteoporosis. After a few weeks to months of minor vertebral fracture, the vertebral collapse and progressive kyphosis gradually occur. The disease is progressive, with intact vertebral bodies or minor fractures in the first stage, mobility instability and vertebral collapse in the second stage, and significant vertebral collapse in the third stage, which can compress the spinal cord and eventually cause paralysis [1]. Therefore, early surgical intervention is recommended for Kümmell's disease.

Percutaneous kyphoplasty (PKP) can provide instant pain relief and strengthen the vertebral body. It has received widespread use in the therapy of first and second Kümmell's disease stages and has produced positive therapeutic outcomes [2, 3]. For patients with neurological deficits, disc decompression surgery should be applied. However, there is no consensus regarding the most effective therapy for Kümmell's disease in its third stage with severe vertebral collapse and no neurological symptoms [4]. Performing PKP for the therapy of Kümmell's disease in Stage III is difficult, according to the conventional view, because of the risk of further collapse and bone cement leakage [5]. Thus, some researchers considered Kümmell's disease Stage III to be a subjective contraindication of PKP. Kümmell's illness in Stage III is characterized by a severely collapsed vertebral body; it can be difficult to recover the vertebral height after resetting the body's position; and an obstructed intravertebral vacuum can hardly provide enough space for bone cement injection and balloon dilatation. Some reports also reported the occurrence of further collapse and cement displacement following PKP. Therefore, open surgery for acute deformity correction through pedicle subtraction osteotomy (PSO) and long‐segment fixation (LSF) has been recommended to treat Kümmell's disease with kyphosis in recent years [6, 7].

It was proposed that the key to a successful result following PKP is to locate a suitable intravertebral vacuum cleft [8, 9]. To solve this issue, a novel vertebral releasing drill was utilized for opening the route through the pedicle, thereby facilitating the utilization of PKP for the therapy for Kümmell's disease in stage III. The internal stabilization created by the vertebral releasing drill can effectively prevent micro‐motion between cement and bone. The objective of this research was to: (i) analyze the clinical and radiological outcomes and complications of PKP and PSO + LSF in the treatment of Kümmell's disease at Stage III without neurological impairments and (ii) evaluate the efficiency and safety of PKP for the reestablishment and decompression of the posterior wall.

## 2 Patients and Methods

### 2.1 Patients

This single‐center, retrospective, and a comparative clinical investigation was approved by the Medical Ethics Committee of Soochow University (ethical code 2020180). All participants provided their informed written permission. From January 2017 to June 2020, a total of 89 (male, 17; female, 72) patients with Kümmell disease in stage III with kyphosis received PKP or PSO + LSF. Forty‐six patients underwent PKP and 43 patients who treated by PSO + LSF. The mean age of the patients was 74.0 ± 8.1 years (range: 61–85 years). The procedures were done by the same surgeon.

Following are the inclusion criteria: (i) patients were identified with Kümmell's disease stage III based on imaging results and clinical symptoms according to the classification of Li et al. [10] and Ma et al. [11], (ii) patients had severe back pain, and spinal cord compression manifested by magnetic resonance imaging (MRI), but no symptomatic neurological deficit existed, (iii) patients with bone mineral density ≤ −2.5 SD; and (iv) patients with kyphosis Cobb's angle ≤ 40°. Exclusion criteria: (i) patients with comminuted bilateral pedicle fracture, (ii) patients with spinal metastases or vertebral tuberculosis that cause pathological vertebral fractures, (iii) Kümmell's disease in Stage III with neurological symptoms, and (iv) patients with hepatorenal dysfunction, severe cardiopulmonary, or previous spinal surgery were excluded from this study.

### 2.2 Surgical Procedures

#### 2.2.1 Percutaneous Kyphoplasty

**FIGURE 1:**
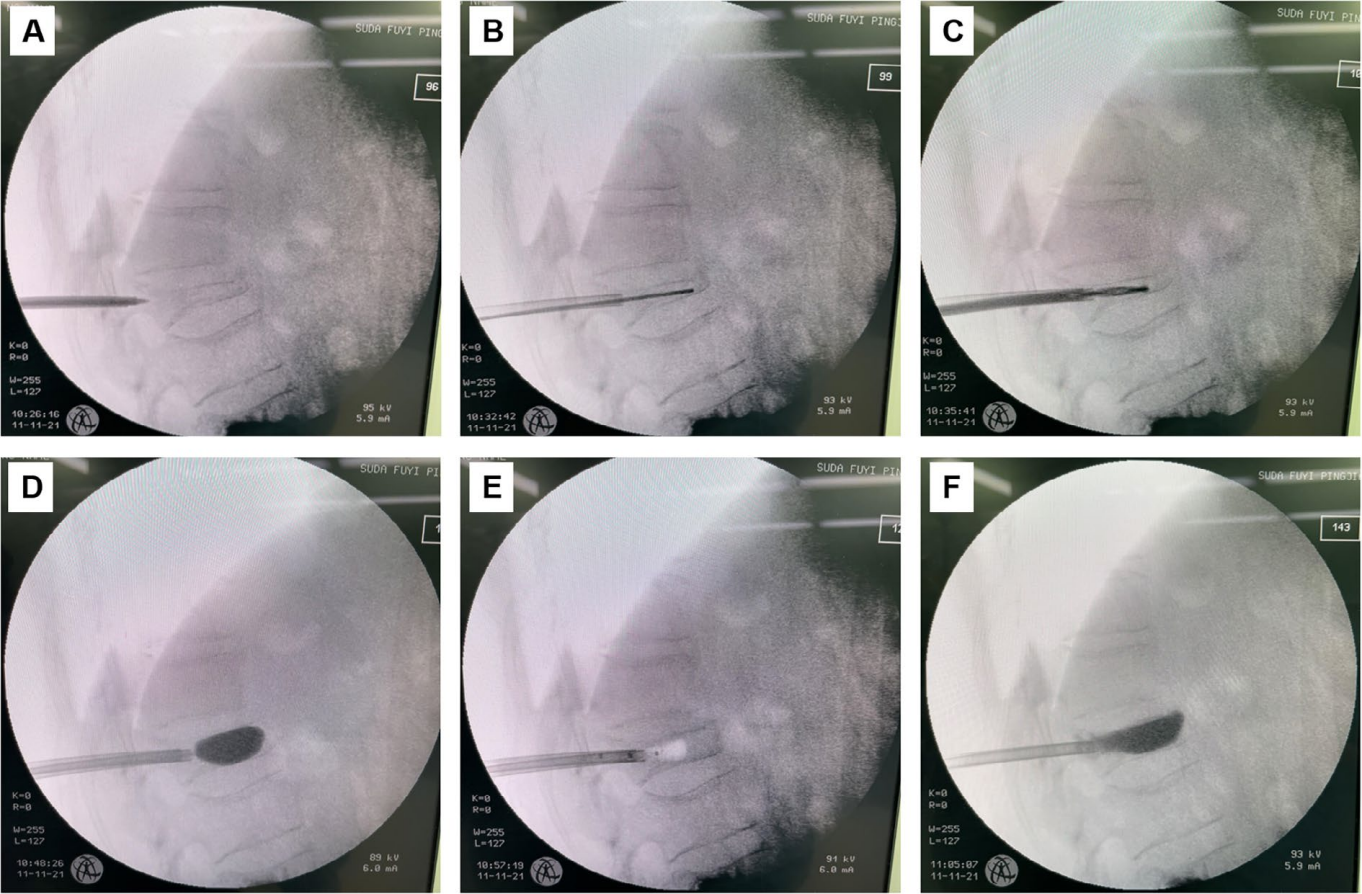
PKP procedure for the treatment of Stage III Kümmell's disease. (A) The vertebral height was not recovered after the body position resetting, so a vertebral releasing drill was used to open the route through the pedicle. (B) The open channel enabled the needles inserted into the fractured vertebral and placed around the cleft of the vertebra. (C) The cutter is enlarged and rotated to release the intravertebral vacuum cleft. (D and E) The height and the Cobb angle of the vertebral were restored after the withdrawal of the bilateral balloons. (F) The cavity was filled and no bone cement leakage was observed under continuous guidance of C‐arm.

General anesthesia was administered to patients in the PKP group, who were then positioned in the prone position with pillows under their waist and upper chest to encourage hyperextension. For those patients whose vertebral height could not be recovered after body position resetting, we have innovated a new surgical method by using a special vertebral releasing drill to open the route through the pedicle (Figure S1). The opened channel enabled the needles inserted into the fractured vertebral and placed around the cleft of the vertebra. The cutter is enlarged and rotated to release the intravertebral vacuum cleft. Bilateral transpedicular working channels were penetrated under CArm Xmedical equipment guidance. Anteroposterior and lateral film was recorded to verify the efficiency and safety of the needles inserted into the fractured vertebral. First at 1/3 of the distance from the posterior wall, then continued to inserted to the anterior 1/4 of the vertebral body, a cutter is enlarged and rotated to release the intravertebral vacuum clefts. Later, bilateral balloons were inserted into the intravertebral vacuum cleft to reestablish the height of the vertebral body and to treat kyphosis. Polymethylmethacrylate (PMMA) bone cement (approximately 3.0 mL) was carefully injected to the point at which it became doughy, and the amount of cement was filled in the cavity (Figure 1). For those patients with the collapse of the anterior wall, middle‐late period PMMA should be injected in 1/3 of the anterior part to block the posterior wall's collapse, with the following injection of the early‐period cement to provide sufficient bone incorporation of the cement.

#### 2.2.2 PSO Combined With Long‐ Segment Fixation (PSO + LSF)

Under general anesthesia, the spine was exposed with a standard midline approach. Pedicle screws were introduced three levels above and three levels below the fracture level after exposing the spinous processes, the fracture level, the adjacent segment, the transverse process, and the facet joint. The spinal canal was laterally opened following the removal of posterior components such as the spinous process, adjacent facet joints, and bilateral lamina at the apical vertebral level. The vertebral body's posterior portion became empty after these procedures. Next, osteotomy with pedicle removal was performed. Two‐thirds of the top vertebral pedicle of the injured vertebrae was removed utilizing a periosteal elevator and sponge stick, revealing the vertebral body's lateral cortex. Using a curette, the upper portion of the body of the vertebrae was excised. Next, the lateral cortex of the vertebrae was osteotomized to enable the osteotomy's closure. A temporary stabilizing rod was inserted to prevent the spinal column from suddenly translating after the osteotomy was finished on one side. A comparable procedure was carried out in the fractured vertebra on the contralateral side. After the PSO was completed, the operating bed was adjusted to allow for closure of the osteotomy. All processes above are performed under C‐arm guidance and neuromonitoring.

### 2.3 Data Collection and Outcome Assessment

Radiologic and clinical data were collected, and all surgeries were carried out by the same surgeon, to investigate the effectiveness of PKP and PSO + LSF in the therapy of Kümmell's disease at Stage III.

Oswestry disability index (ODI) was utilized to assess the impact on the patient's daily existence. Using a visual analog scale (VAS), the impact of the patient's back discomfort was determined. Additionally, the ODI recovery rate (RR) was determined. RR = (preoperative ODI − postoperative ODI)/(preoperative ODI) * 100%. Kyphotic Cobb's angle was measured in the standard lateral radiograph by drawing a straight line from the inferior endplate one level below the fractured body and the superior endplate one level upper the fractured body. In a lateral X‐ray, the central and anterior heights of the fractured body were assessed. The ratio of vertebral height = (height of fractured body)/(mean height of adjacent vertebra body) * 100%. After surgery, at 3 days, 1, 3, 6, and 12 months, as well as every year after that, data on the ODI, VAS, Cobb's angle, central and anterior vertebral heights were collected. In addition, the duration of the surgical procedure, length of stay (LOS), complications, and estimated blood loss were documented and evaluated.

### 2.4 Statistical Analysis

All evaluations were conducted using IBM SPSS 19.0 (NY, USA). Mean values and standard deviations were calculated. The independent *t*‐test was implemented to compare preoperative and postoperative continuous factors between groups. Fisher's exact test or chi‐square test for number and percentage data was employed to compare statistical analyses between two groups. *p* values less than 0.05 were statistically significant.

## 3 Results

### 3.1 Demographics and Perioperative Outcomes

All PKP and PSO + LSF surgeries were successfully performed, and the follow‐up period ranged from 24 to 48 m (mean 30.8 ± 6.8 m). Patients in the PKP and PSO + LSF groups did not vary significantly with respect to sex, age, and bone mineral density (BMD). The PKP group experienced considerably less blood loss (17.0 ± 8.4 mL) than the PSO + LSF group (1088 ± 130 mL; *p* < 0.05). Compared with the PKP group, the PSO + LSF group required a longer operation time (42.1 ± 6.3 min for PKP vs. 240 ± 22 min for PSO + LSF; *p* < 0.05), and needed a longer LOS (5.8 ± 1.6 days for PKP vs. 15 ± 3.8 days for PSO + LSF; *p* < 0.05). Patient demographics are summarized in Table 1.

**TABLE 1:** General data of patients in two groups.

	PKP	PSO + LSF	*p*
Patients number	46	43	
Gender (male/female)	8/38	9/34	0.789
Age (years)	73.6 ± 7.1	74.5 ± 6.9	0.557
Operative time (min)	42.1 ± 6.3	240 ± 22	0.000
Estimated blood loss (mL)	17.0 ± 8.4	1088 ± 130	0.000
LOS (days)	5.8 ± 1.6	15 ± 3.8	0.000
BMD (T score)	−3.31 ± 0.5	−3.40 ± 0.7	0.495

### 3.2 Clinical Outcomes

The mean ODI score and VAS score decreased significantly from pre‐operation to the final follow‐up in both groups (*p* < 0.05). In the PKP group, the ODI and VAS scores showed no significant difference when comparing the final follow‐up data and those immediately after the operation. However, the ODI and VAS scores in the PSO + LSF group substantially decreased at the final follow‐up compared to 3 days after surgery. The ODI RR for the PKP and PSO + LSF groups at the last follow‐up was 62.2 ± 11.3 and 60.3 ± 8.3, respectively. The PKP group scored significantly lower than PSO + LSF in ODI, VAS, and RR at 3 days and at 3 months postoperatively, but no significant difference was found in both ODI and VAS between PKP and PSO + LSF group at the final follow‐up. The data of the two groups are summarized in Table 2.

**TABLE 2:** Clinical outcomes between PKP and PSO + LSF.

Variables	PKP	PSO + LSF	*p*
ODI scores			
Preoperative	76.6 ± 10.3	77.4 ± 9.8	0.719
Postoperative 3 days	34.6 ± 9.4	69.2 ± 10.2	0.000
postoperative 3 months	31.8 ± 10.1	51.1 ± 7.7	0.000
Final FU	29.0 ± 4.2	30.7 ± 5.9	0.123
VAS scores			
Preoperative	8.2 ± 0.7	8.0 ± 0.9	0.255
Postoperative 3 days	2.0 ± 1.0	5.5 ± 0.8	0.000
Postoperative 3 months	1.8 ± 1.1	3.9 ± 1.2	0.000
Final FU	1.9 ± 1.0	1.7 ± 1.1	0.331
RR (%)			
Postoperative 3 days	39.5 ± 14.4	10.6 ± 13.5	0.000
Postoperative 3 months	59.1 ± 9.9	34.2 ± 9.4	0.000
Final FU	62.2 ± 11.3	60.3 ± 8.3	0.369

### 3.3 Radiologic Outcomes

**FIGURE 2:**
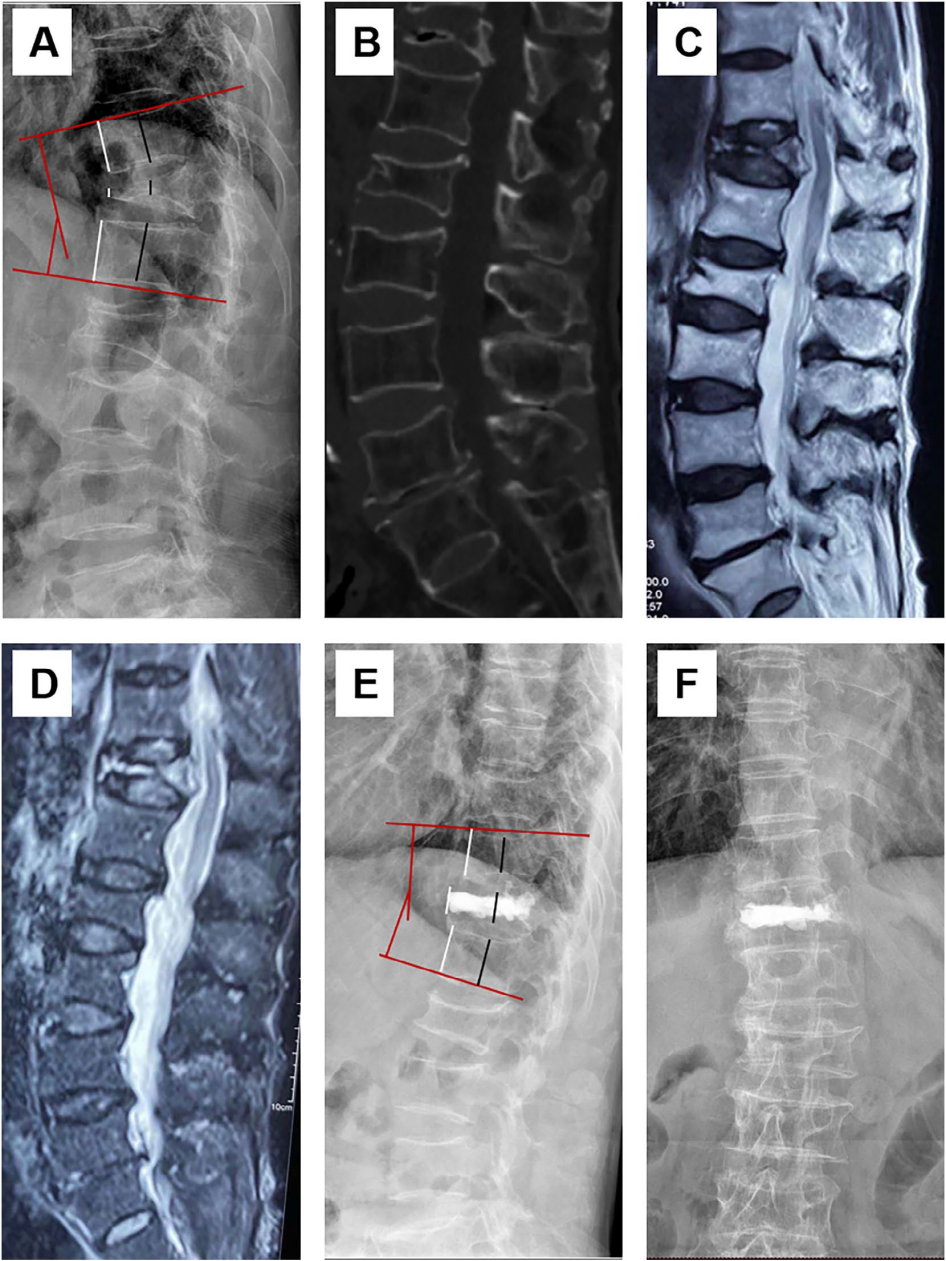
A 72‐year‐old female patient, with back pain for 6 months, who underwent PKP. (A) Preoperative radiograph, manifesting the fracture vertebral with serious collapse. The Cobb angle was 22°, the ratio of central and anterior vertebral height was 26.5% and 16.3%, respectively. (B) Preoperative CT scan showed T12 vertebral fracture with intravertebral cleft and collapsed posterior wall. (C) T2‐weighted image showed spinal compression. (D) STIR image showed high signal of the necrotic area. (E and F) Postoperative X‐ray: The Cobb angle recovered to 9°, the ratio of central and anterior vertebral height recovered to 75.2% and 57.9%, respectively.

**FIGURE 3:**
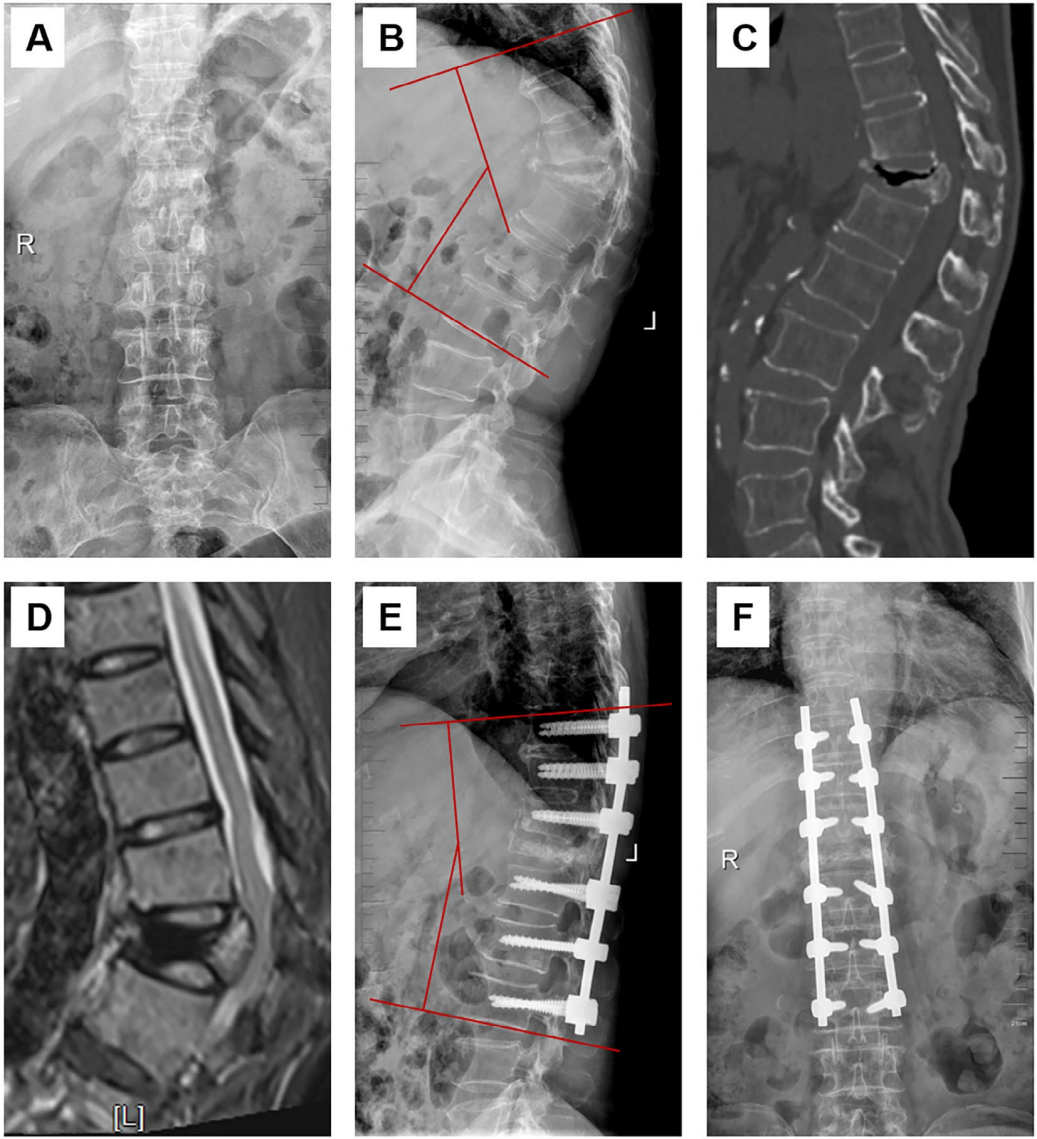
Female patient, aged 68 years, with kyphosis and back pain for 9 months, who underwent PSO + LSF. (A–D) Preoperative images show the vertebra was extremely compressed, the Cobb angle was 50.5°, and T2‐weighted image showed the obvious spinal compression. (E and F) The Cobb angle recovered to 14° and satisfactory radiographic results on postoperative X‐ray images.

There was no significant difference in preoperative Cobb angle between the two groups (30.4 ± 13.0 for PKP vs. 34.3 ± 15.9 for PSO + LSF, *p* > 0.05). Compared with the preoperative values, the kyphotic Cobb angle was significantly corrected at the final follow‐up in both groups (*p <* 0.05). In addition, the PSO + LSF group exhibited better outcomes in kyphotic Cobb angle correction than the PKP group at the final follow‐up (14.1 ± 5.8 for PKP vs. 10.4 ± 4.3 for PSO + LSF, *p <* 0.05). The ratio of central and anterior vertebral height was significantly improved from pre‐operation to post‐operation at the final follow‐up in the PKP group (*p <* 0.05). The radiological data of patients in both groups are shown in Table 3. Illustrative cases are shown in Figures 2 and 3.

**TABLE 3:** Radiographic outcomes between PKP and PSO + LSF.

Variables	PKP	PSO + LSF	*p*
Kyphotic Cobb angle (°)			
Preoperative	30.4 ± 13.0	34.3 ± 15.9	0.211
Postoperative 3 days	15.8 ± 3.0	10.9 ± 5.3	0.000
Postoperative 3 months	13.4 ± 4.6	9.7 ± 4.8	0.000
Final FU	14.1 ± 5.8	10.4 ± 4.3	0.000
The ratio of central vertebral height (%)			
Preoperative	35.2 ± 7.2	34.5 ± 6.3	0.628
Postoperative 3 days	69.1 ± 4.4	21.8 ± 5.9	0.000
Postoperative 3 months	70.2 ± 5.3	22.3 ± 6.2	0.000
Final FU	70.5 ± 4.2	22.7 ± 4.5	0.000
The ratio of anterior vertebral height (%)			
Preoperative	29.3 ± 8.1	28.2 ± 7.4	0.526
Postoperative 3 days	68.3 ± 4.4	29.0 ± 5.3	0.000
Postoperative 3 months	69.8 ± 5.1	29.3 ± 4.1	0.000
Final FU	68.8 ± 5.4	30.5 ± 4.5	0.000

### 3.4 Complications

In the PKP group, five patients had asymptomatic cement leakage, including three anterior‐vertebral cases, one intervertebral case, and one paravertebral case. In the PSO + LSF group, there was one dural tear recognized and repaired during operation. Two cases of screw loosening, but in all cases the screw loosening did not progress and the X‐ray showed a solid fixation. There was one who experienced delayed wound healing and one who contracted pneumonia in the PSO + LSF group, and all the patients recovered following conservative treatment.

## 4 Discussion

### 4.1 Main Findings of This Study

In the present study, 89 patients of Kümmell's disease at Stage III without neurological impairments were treated with PKP or PSO + LSF. Postoperative measurements showed substantial improvements in kyphosis Cobb angle and vertebral height in the PKP group compared to preoperative measurements. Operating time, estimated blood loss, and duration of stay were all reduced in the PKP group compared to the PSO + LSF group. The PSO + LSF group had better correction of a kyphotic Cobb angle than the PKP group. Short‐term monitoring showed that the PKP group had fewer ODI and VAS scores than the PSO + LSF group. Kümmell's disease in Stage III without neurological symptoms responded to both PKP and PSO + LSF as safe and efficient treatments.

### 4.2 Other Newly Reported Surgical Techniques for Kümmell's Disease

Kümmell's disease is more prevalent following OVCF with vertebral body vascular osteonecrosis. Conservative therapy, including medical pain control, bed rest, and analgesics, was ineffective for Kümmell's disease because of its progressive nature. It might result in spinal canal stenosis, neurological deficits, or chronic lower back discomfort [12, 13]. Without surgery, the vertebra leads to nonunion and collapse, which eventually results in paralysis [14]. Therefore, surgical intervention is sometimes recommended for Kümmell's disease.

According to the clinical classification, there are three stages to Kümmell's disease [11]. The posterior wall of the damaged vertebra stays intact in the first and second stages of Kümmell's disease, making PKP and vertebroplasty (VP) an effective treatment option [15]. Because it can immediately relieve pain, correct kyphosis, restore the height of the vertebral volume, and accomplish vertebral stability [16]. Stage III Kümmell's disease, however, is characterized by a complete collapse of the posterior wall, which results in intense back pain, and progressive kyphosis, and may or may not be accompanied by neurological dysfunction [17]. For Kümmell's disease in Stage III with neurological deficits, the benefits of open therapies include adequate decompression and solid fixation for the attainment of intravertebral stability. There is currently no defined plan of action and no single efficient therapy for Kümmell's disease Stage III in patients without neurological deficits. Traditional surgical methods, including anterior and posterior surgery, are mainly vertebral canal depression, PSO, internal fixation, and orthopedic bone grafts [18, 19]. According to a study, anterior expandable strut cages can be employed to restore the stability of the anterior column, but there are some clear drawbacks, including the risk of implant sinking that could progress, postoperative atelectasis, and high levels of surgical trauma [20]. Lu et al. compared PKP and short‐segmental fixation combined with vertebroplasty and concluded that both methods are safe and effective for Kümmell's disease, while PKP can shorten the operation time and reduce the volume of blood loss [9]. Zhang et al. put up with PSO combined with LSF for the treatment of Kümmell's disease in its third stage, and they report that PSO + LSF provides both decompression of the spinal cord and sagittal correction of kyphotic deformity [6]. Other strategies, such as posterior vertebral column resection, posterior fixation combined with vertebroplasty, and posterior spinal shortening osteotomy, may also affect functional recovery and early pain relief [21, 22]. As Kümmell's disease often occurs in elderly patients older than 50 years, complex surgical methods such as anterior and posterior approaches produce a high rate of internal fixation failure due to the severity of osteoporosis and comorbidities. Therefore, a complicated open operation may not be the best option for Stage III Kümmell's disease in the absence of neurological symptoms. As a minimally invasive procedure, PKP is characterized by low risk of complications, high benefits in terms of slight trauma, low blood loss, short operating times, cheap costs, and favorable postoperative results. Also, PKP avoids a series of complications such as contracted pneumonia, nerve damage, and deep vein thrombosis caused by a long time in bed [4, 23].

### 4.3 The Feasibility of PKP in the Treatment of Stage III Kümmell's Disease

PKP and PVP are frequently recommended by researchers as successful methods to treat Kümmell disease Stages I and II. However, due to the incomplete posterior body wall of the vertebrae, the trouble of puncture, and the high rate of bone cement leakage, many surgeons believed it to be a relative contraindication to use PKP or PVP in treating Kümmell's disease in its third stage [24, 25]. According to Xia et al., PKP may be an option for treating Kümmell's disease. All of them demonstrated a substantial reduction in low back pain, kyphotic Cobb angle, and vertebral height [26]. Lee et al. studied 10 Kümmell's disease patients who received PKP in conjunction with short‐segment fixation in 2011. PKP has the potential to lessen the amount of blood lost and shorten the duration of the surgery [27]. However, there are still some PKP issues that require further investigation, and there are very few reports on the use of PKP to treat Kümmell's disease in Stage III [4]. According to research, incomplete posterior vertebral body walls and insufficient bone cement augmentation will result in high rates of bone cement leakage and bone displacement during PKP therapy for Kümmell's disease [28, 29].

### 4.4 Our Experience in PKP Treating Stage III Kümmell's Disease Without Neurologic Symptoms

Based on the quantity of clinical practice, PKP was considered a viable option for treating Kummell's disease in Stage III patients who did not exhibit neurologic symptoms. Importantly, an intravertebral vacuum cleft is the PKP technique's basis for achieving a favorable outcome for treating Kummell's disease in Stage III [30]. Stage III Kümmell's disease manifests as severe posterior body collapse, an incomplete posterior vertebral body wall, or compression of the corresponding section of the vertebral body. Therefore, it was difficult to locate an intravertebral vacuum cleft, and it was also challenging to create sufficient space for bone cement injection and balloon dilatation. As a result, the therapy of Stage III Kümmell's disease with the PKP approach was hampered.

We explored a two‐step approach to solve this problem. The patient was initially positioned in a hyperextended posture to encourage the reduction of the diseased vertebrae. A tractive impulse from the anterior longitudinal ligament and intervertebral disc may now cause the cleft to widen in some patients, whose vertebral height has partially recovered. Second, for those patients whose vertebral height could not be recovered after body position resetting, we have innovated a new surgical method by using a special vertebral releasing drill to open the route through the pedicle. The opened channel enabled the needles inserted into the fractured vertebral and placed around the cleft of the vertebra. The cutter is enlarged and rotated to release the intravertebral vacuum cleft. Following that, the intravertebral vacuum cleft allowed for proper balloon dilation and positioning, restoring the Cobb angle and height of the fractured vertebrae. It also created the necessary conditions for bone cement to be filled under low pressure with a high viscosity, preventing cement leakage. For those patients with the collapse of the anterior wall, middle‐late period PMMA should be injected in 1/3 of the anterior part to block the posterior wall's collapse, with the following injection of the early‐period cement to provide sufficient bone incorporation of the cement. The continuous C‐arm guidance allowed for the observation of the bone cement injection, which needs to be prevented as soon as a leak appears. The posterior longitudinal ligament's force successfully reduced and tightened the posterior wall after being in the prone position. Therefore, the injection of bone cement was immediately halted when the cement spread to the collapsing posterior wall. In addition, it is essential to monitor the quantity of bone cement due to the leakage induced by excessive bone cement. According to observations, a small quantity of bone cement in the middle‐late phase should be injected first to seal the anterior wall's defects before the early phase bone cement. When the cement injection is slightly greater than balloon dilation, the volume of bone cement is appropriate. According to the findings of this study, asymptomatic bone cement leakage was less than previously reported [31]. For those patients who have diagnosis with osteoporosis, combination therapy of anabolic (e.g., teriparatide) and anti‐resorptive agents (e.g., bisphosphonates, denosumab, calcium, and vitamin D) is expected to be an ideal anti‐osteoporosis option. Patients who underwent PKP and PSO + LSF started their back muscle rehabilitation exercises at 1‐week post‐operation when patients' walking ability was regained.

Both PKP and PSO + LSF were shown to be effective and safe treatments for patients with Stage III Kümmell's disease who exhibited no neurological signs in this investigation. Although PSO + LSF may be the better choice for patients with severe kyphosis, PKP is still characterized as a less invasive, lower risk for complications, and effective method for alleviating pain. The VAS and ODI scores in the PKP group were considerably lower than those in the PSO + LSF group when comparing immediate postoperative outcomes, but there was no significant difference between the two groups' long‐term effects. The possible reason is due to the PKP procedure causes less trauma, lower blood loss, and operation time, which can affect immediately after surgery with respect to the functional recovery and early pain relief. This is especially important for adults older than 50 years with comorbidities who cannot tolerate complex surgical methods. In addition, the medical cost of PKP is less than that of PSO + LSF. In the prior research, internal fixation procedures had a higher risk of complications than PKP, but this study's analysis found no discernible difference in complications between the two groups [8]. Furthermore, a mild reduction in kyphosis correction was observed in both PKP and PSO + LSF groups at the final follow‐up, but there is no significant difference.

### 4.5 Limitations and Strengths of This Study

The current investigation's limitations include both the retrospective nature of the research and the lack of any technique for ensuring unbiased randomization between the two groups. Due to the exclusion of patients with neurological deficits, it is inadequate to show the effectiveness of PKP for the reestablishment and decompression of the posterior wall. Also, there is no unified classification for Kümmell's disease, we will find the availability of other extensive classification systems to describe fracture morphology in detail. In further study, we will provide more meaningful comparison, for example, PKP vs. PKP + short segment fixation or PKP with fixation vs. PSO + LSF. Additionally, more cases and an extended period of follow‐up are required to assess the clinical results of PKP and PSO + LSF for the therapy of Kümmell's disease in Stage III.

## 5 Conclusions

According to this investigation, Kümmell's disease in its Stage III without neurological symptoms can be treated effectively with both PKP and PSO + LSF. The study found that PKP can efficiently and safely reduce pain in Kümmell's disease patients. In addition, PKP was associated with early clinical outcomes, quicker pain relief, less surgical trauma, and decreased blood loss than PSO + LSF.

## Author Contributions

**Yijie Liu:** conceptualization, data curation, investigation, methodology, writing – original draft. **Tangyiheng Chen:** data curation, formal analysis, investigation. **Haoyun Yu:** formal analysis, writing – review and revision. **Xiaohui Zhou:** formal analysis, investigation. **Runjia Hua:** data curation, formal analysis. **Yudong Wang:** data curation, formal analysis. **Qiang Wei:** formal analysis, investigation. **Yong Gu:** data curation, methodology, resources, writing – review and revision. **Genglei Chu:** conceptualization, data curation, formal analysis, funding acquisition, investigation, methodology, resources, writing – original draft, writing – review and revision.

## Ethics Statement

This study was performed in line with the principles of the Declaration of Helsinki. Approval was granted by the Ethics Committee of Soochow University (ethical code 2020180). Informed consent was obtained from all individual participants included in the study.

## Consent

The authors have nothing to report.

## Conflicts of Interest

The authors declare no conflicts of interest.

## Supporting information


**Data S1.** Supporting Information.

## Data Availability

The data sets used and/or analyzed during the current study are available from the corresponding author on reasonable request.
