# Supplementary material for: Is Percutaneous Kyphoplasty the Better Choice for Treatment of Stage III Kümmell's Disease Without Neurological Symptoms? A Retrospective Study of Two Invasive Procedures
Source: Orthop Surg. 2024 Dec 16;17(3):724–32. doi: 10.1111/os.14313 (PMC11872362; doi:10.1111/os.14313)
Supplement: Supplementary file 1 — Data S1. Supporting Information. [file OS-17-724-s001.docx]

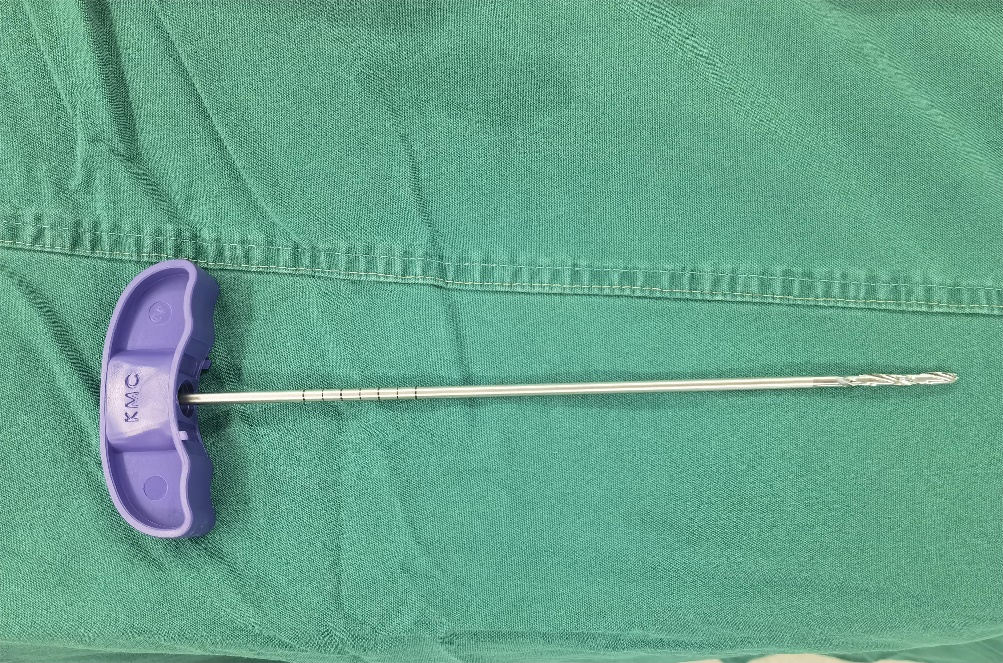


**Figure S1**. A vertebral releasing drill to open the route through the pedicle for those patients whose vertebral height could not be recovered after body position resetting.
